# *Rhizopus homothallicus,* an emerging pathogen causing cavitary lung lesions

**DOI:** 10.1099/acmi.0.000526.v3

**Published:** 2023-04-21

**Authors:** Juhi Taneja, Kuhu Chatterjee, Ruchi Arora Sachdeva, S. Zafar Abbas, M.K. Sen

**Affiliations:** ^1^​ Department of Microbiology, ESIC Medical College and Hospital, Faridabad, Haryana, India; ^2^​ Department of Respiratory Medicine, ESIC Medical College and Hospital, Faridabad, Haryana, India; ^3^​ Department of Radiology, ESIC Medical College and Hospital, Faridabad, Haryana, India

**Keywords:** mucormycosis, *Rhizopus homothallicus*, cavitary, pulmonary

## Abstract

**Introduction.:**

*Rhizopus homothallicus* is an emerging pathogen that causes pulmonary mucormycosis.

**Case Presentation.:**

We report a case of pneumonia caused by *R. homothallicus* in a 54-year-old type 2 diabetic patient. The organism was isolated from bronchoalveolar lavage fluid and preliminarily identified by fungal morphology and finally by sequencing of the internal transcribed spacer region.

**Conclusion.:**

Mucormycosis may be associated with cavitary lung lesions against a backdrop of poorly controlled diabetes or other immunosuppressed states. Pulmonary mucormycosis may have variable clinical and radiological presentations. Therefore, strong clinical suspicion and prompt management can address the high fatality associated with the disease.

## Data Summary

The following ITS sequence has been submitted to GenBank under accession number OP019829:

1 ttctcgcatc gatgaagaac gtagcaaagt gcgataatag tgtgaattgc atattgtgaa

61 tcatcgagtc tttgaacgca gcttgcactc tatggttctt ccatagagta cgcctgcttc

121 agcatcataa caatcccaca cataaaaatt tttttttatg tggttatggg caattctttc

181 atagtatgga atcgcctaaa aaattctagg tataggtgct tgaataaagg atcatatctc

241 caatccattt tttgggagac caaggaaaca ggattgggcc accgacatac cctcatatat

301 tctgaagt caggtgggac tacccgctga acttaagcat atcaataag

## Introduction

Ubiquitous fungi such as *Rhizopus*, *Absidia*, *Rhizomucor*, *Mucor* and *Cunninghamella*, species of the order Mucorales, are found in the soil and cause opportunistic infections. The known predisposing factors in patients with mucormycosis are uncontrolled diabetes, neutropenia, carcinomas, immunosuppressive therapy, deferoxamine therapy and voriconazole prophylaxis [1, 2]. Among the genera Rhizopus, *Rhizopus homothallicus* is being reported in a large number of cases and is an emerging pathogen [1]. The sporangiospores released by Mucorales gain entry to the upper or lower airways through aerosolization. Pulmonary mucormycosis is second in frequency after rhino-orbital-cerebral among the reported cases of mucormycosis.

## Case Presentation

**Fig. 1.**
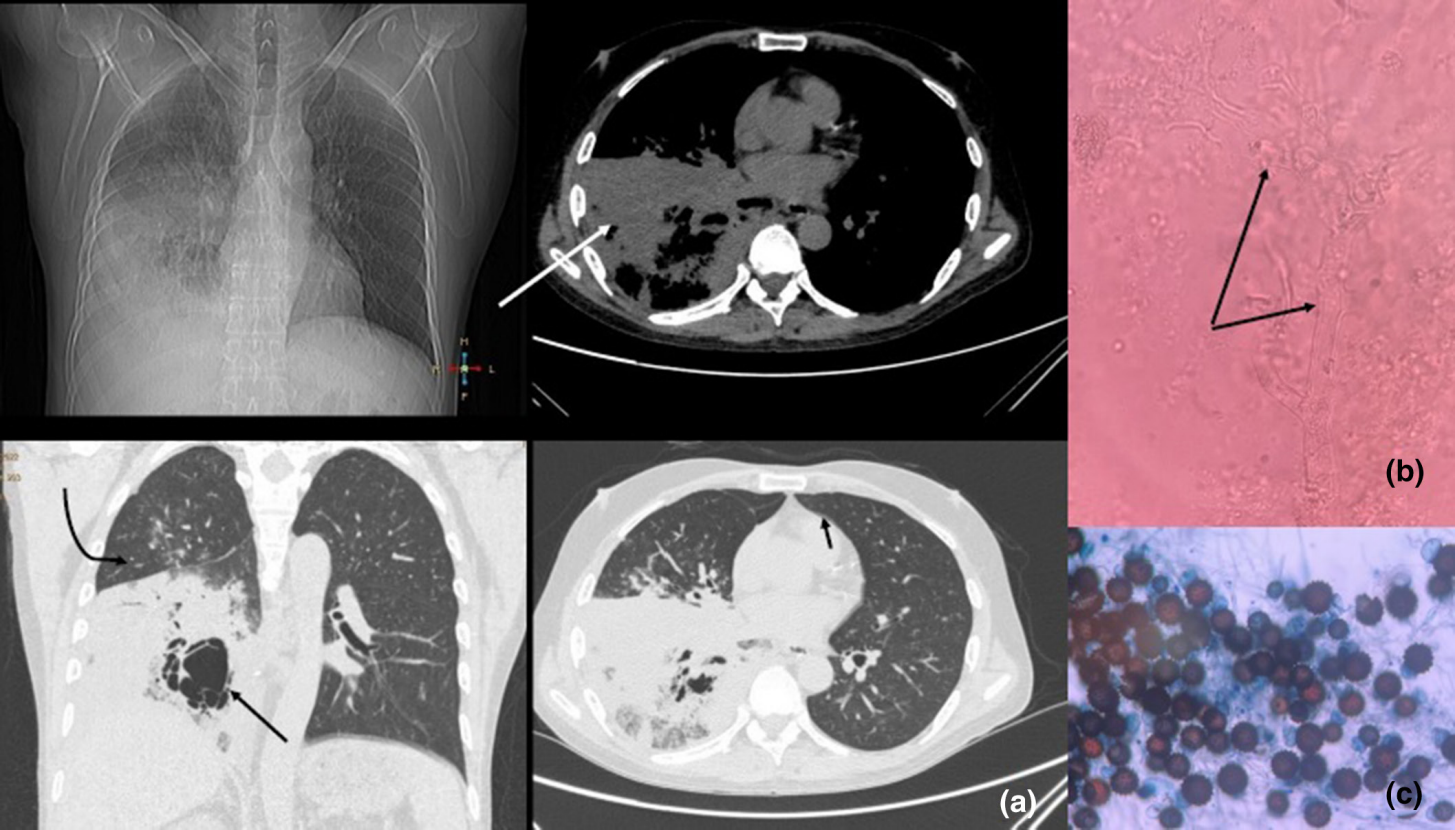
(a) Chest radiograph (first spot) showing non-homogenous opacification of right mid-lower zone with air bronchogram and obscured right costophrenic angle and right hemidiaphragm s/o Lobar pneumonia. HRCT of the chest with mediastinal and lung windows (second to fourth spots) showing dense opacification of right lower lobe (white arrow) limited by horizontal fissure s/o lobar pneumonia with multiple irregular cavities within it (long black arrow). Patchy area of consolidation seen in right upper lobe on coronal image (curved black arrow). (b) KOH mount of bronchoalveolar lavage, with arrows showing broad non-septate hyphae, which gives presumptive evidence for mucormycosis. (c) Lactophenol cotton blue (LPCB) mount showing *R. homothallicus* with characteristic golden brown, globose zygospores with stellate spines and suspensor cells (100×).

A 54-year-old male with type 2 diabetes mellitus presented with complaints of intermittent fever, cough with expectoration, occasional haemoptysis and right-sided chest pain for the previous 20 days. There was no history of coronavirus disease 2019 (COVID-19) infection in the recent past. The patient tested negative for severe acute respiratory syndrome coronavirus 2 (SARS-CoV-2) by RT-PCR and gave a history of vaccination with two doses of Covishield. The patient was a known case of type 2 diabetes mellitus for the previous 20 years and was on irregular medication of metformin and pioglitazone. On examination, the patient was afebrile, conscious and oriented. On auscultation, breath sounds were markedly reduced on the right side with fine crepitations. Renal function, liver function tests and urinalysis were unremarkable except for blood sugar levels of 516 mg dl^−1^ and total leucocyte count of 13 000 mm^−3^ on admission. Testing for HbA1c level was not performed. There was no evidence of acid-fast bacilli in the sputum. A serum galactomannan test was negative. Chest X-ray revealed consolidation in the middle and lower zone of the right lung (Fig. 1a). On day 4 blood parameters showed a markedly elevated total leucocyte count (>35 000 mm^−3^) with neutrophilia (>90 %) and lymphocytopenia (~3 %), and high procalcitonin (15.3 ng ml^−1^), d-dimer (10 000 ng ml^−1^), creatinine (4 mg dl^−1^), urea (200 mg dl^−1^), SGOT (209 U l^−1^) and SGPT (167 U l^−1^) levels. The patient also showed traces of ketones in urine and was subsequently shifted to the intensive care unit for close monitoring.

On admission the patient was initiated on amoxicillin/clavulanic acid, and clarithromycin. High-resolution computed tomography (HRCT) chest performed on day 2 showed a large area of consolidation with bronchiectasis, fibrosis and secondary cavitary changes showing air–fluid levels in the right lower lobe with intra- and interlobular septal thickening with ground glass haze giving a crazy paving pattern in the right lower lobes and a few patchy areas of consolidation in the right upper and middle lobe with minimal right pleural effusion (Fig. 1a). On day 4, as the patient deteriorated clinically with persistent fever, high blood counts and increasing consolidations seen on chest radiograph, the antibiotics were escalated to meropenem and teicoplanin. On day 5 due to further deterioration in the condition of the patient, fungal aetiology was suspected. A bronchoalveolar lavage sample was collected and sent for microbiological analysis. KOH examination was performed, which showed broad aseptate fungal hyphae (Fig. 1b). On day 5, the patient underwent sudden respiratory distress, for which he was intubated. Taking into account the reduced urine output and altered renal function, the patient was initiated on dialysis. The patient was started on posaconazole as the salvage therapy. On admission day 6, the patient died due to sudden cardiopulmonary arrest and could not be revived.

**Fig. 2.**
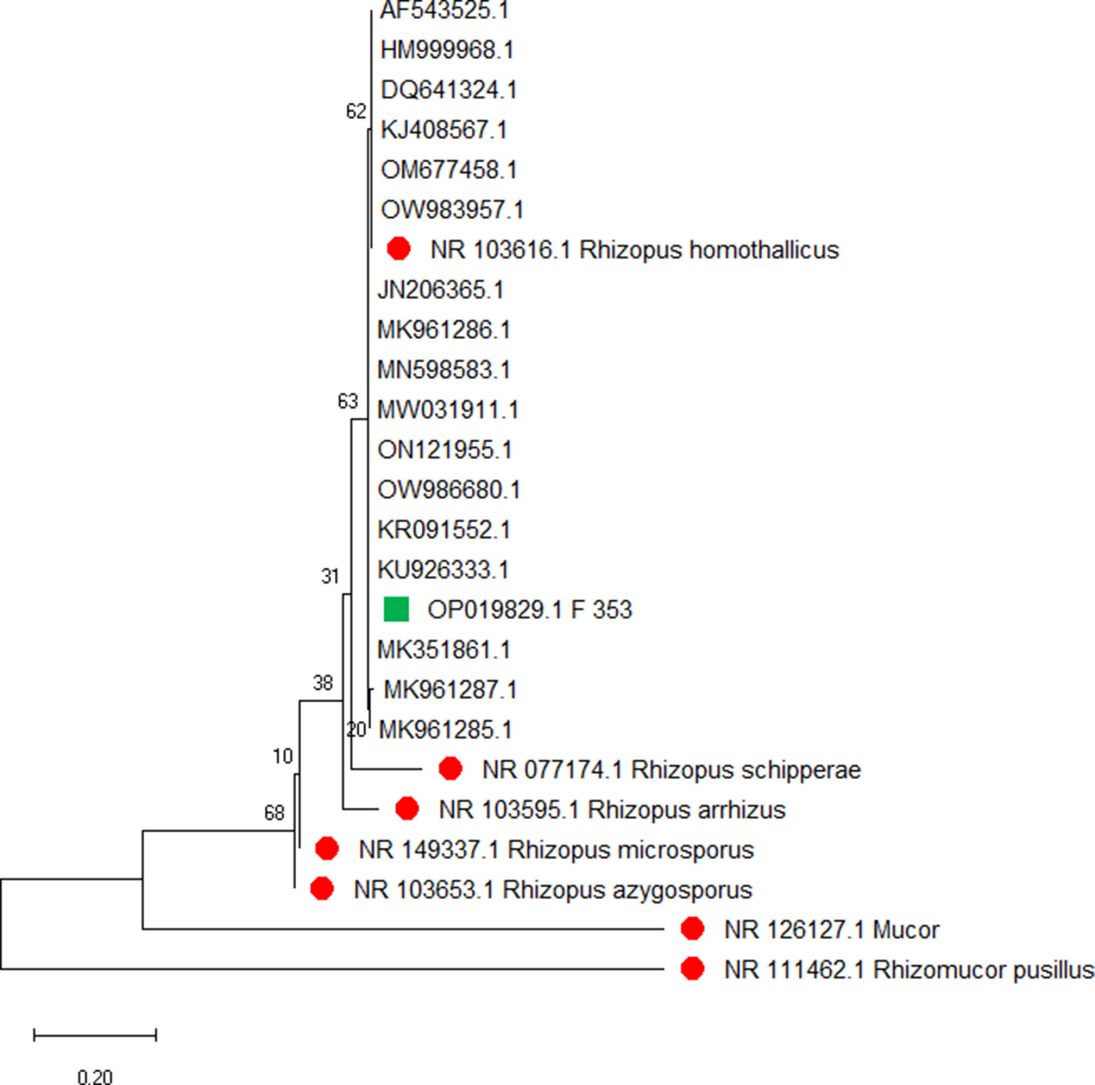
Phylogenetic tree constructed using mega X. The reference strains are represented by red dots and the strain under study is represented by a green square.

Fungal culture results showed a fast-growing, cottony white colony turning brown on Sabouraud dextrose agar (SDA) incubated at 25 °C and 37 °C, within 48 h of incubation. The isolate was identified as *R. homothallicus* based on the characteristic golden-brown spiny zygospores and suspensor cells, along with broad, aseptate hyphae, seen on a lactophenol cotton blue (LPCB) mount (Fig. 1c). DNA was extracted and outsourced for PCR targeting the internal transcribed spacer 2 followed by Sanger sequencing. The sequence has been submitted to GenBank with accession number OP019829. A sequence alignment was performed using the ISHAM database and it was confirmed to be *R. homothallicus* with a similarity index of 96.97 %. The evolutionary tree was prepared using the neighbour-joining method and evolutionary analysis was conducted using mega X (Fig. 2) [3].

## Discussion

*Rhizopus* species are the most commonly isolated fungi from the patients of mucormycosis. *Apophysomyces elegans*, *Apophysomyces variabilis* and *Rhizopus homothallicus* are reported to be emerging species [4]. Chakrabarti *et al*. first reported infections due to *R. homothallicus* in pulmonary infection patients [5]. There are published reports of several cases that indicate the geographical niche of the fungi causing pulmonary, cutaneous and rhino orbitocerebral mucormycosis [5–10]. The incidence of the disease is high in India and also rising globally with the rise in cases of diabetes mellitus [11].

Our patient was diagnosed with sepsis and DKA and was at high risk of developing mucormycosis. In diabetic ketoacidosis (DKA), hyperglycaemia and low pH make phagocytes dysfunctional, with impaired chemotaxis and defective intracellular killing of the pathogen [12]. Mucorales possess the unique ability to acquire iron from the host, which enables the pathogens to grow. In DKA patients there is proton-mediated release of ferric ions from transferrin [12]. Another mechanism has been identified through which there is DKA-enhanced upregulation of glucose-regulated protein (GRP78), which acts as a receptor that mediates entry and damage of endothelial cells by Mucorales [13]. The growth of the *Rhizopus* in the body causes extensive vessel thrombosis and tissue necrosis, as a result of which the fungus disseminates to other organs [12].

Pulmonary mucormycosis has been described in patients with associated immunosuppression, such as neutropenia or graft-versus-host disease, whereas rhino-orbital disease is typically reported in patients with uncontrolled diabetes [14]. The patient in the present setting had longstanding poorly controlled diabetes and presented with DKA. Radiology is considered to be a sensitive marker of pulmonary fungal infection, and the presence of the reverse halo sign on computed tomography (CT) scans has been suggested to be a strong indicator of pulmonary mucormycosis [15]. The present case presented with cavitary pneumonia, which is a rare finding [5]. The clinical findings and chest imaging features are not specific, hence pulmonary mucormycosis is easily misdiagnosed, which can result in serious consequences.

Microscopy and culture of the bronchoalveolar lavage fluid remain the gold standard to establish a diagnosis of pulmonary mucormycosis. The first-line drug is liposomal amphotericin B; however, the clinical team, on assessing the risk/benefit in view of the nephrotoxicity of the drug, initiated posaconazole. The patient succumbed to the disease due to delay in reaching to the healthcare facility and also a delay in initiating antifungal treatment, as fungal infection was not suspected at the outset. Early surgical treatment, appropriate antifungal therapy, and control of predisposing factors are of great importance in the treatment of such cases [16]. However, as patients with pulmonary mucormycosis deteriorate rapidly, the overall mortality rate reported is 40–76 % [17].

The internal transcribed spacer sequence showed maximum similarity to sequences KU926333 and MK351861, as shown in Fig. 2. MK351861 was isolated from soil at Ratapani wildlife sanctuary in Raisen, Madhya Pradesh [18]. Sequence KU926333 was isolated from the lung biopsy sample of a fatal case of pulmonary mucormycosis in a patient with unchecked diabetes mellitus from Paris [19]. Genomic identification of the fungi is strongly recommended for improved epidemiological understanding of mucormycosis.
